# A Dynamic Model of Human and Livestock Tuberculosis Spread and Control in Urumqi, Xinjiang, China

**DOI:** 10.1155/2016/3410320

**Published:** 2016-07-25

**Authors:** Shan Liu, Aiqiao Li, Xiaomei Feng, Xueliang Zhang, Kai Wang

**Affiliations:** ^1^Department of Public Health, Xinjiang Medical University, Urumqi 830011, China; ^2^Urumqi Animal Disease Control and Diagnosis Center, Urumqi 830063, China; ^3^Department of Mathematics, Yuncheng University, Yuncheng 044000, China; ^4^Department of Medical Engineering and Technology, Xinjiang Medical University, Urumqi 830011, China

## Abstract

We establish a dynamical model for tuberculosis of humans and cows. For the model, we firstly give the basic reproduction number *R*_0_. Furthermore, we discuss the dynamical behaviors of the model. By epidemiological investigation of tuberculosis among humans and livestock from 2007 to 2014 in Urumqi, Xinjiang, China, we estimate the parameters of the model and study the transmission trend of the disease in Urumqi, Xinjiang, China. The reproduction number in Urumqi for the model is estimated to be 0.1811 (95% confidence interval: 0.123–0.281). Finally, we perform some sensitivity analysis of several model parameters and give some useful comments on controlling the transmission of tuberculosis.

## 1. Introduction

Tuberculosis (TB) is a worldwide public health problem that is chronic infectious disease of respiratory tract as the main route of transmission. In 1993, WHO declared a state of the global TB in emergency. Even if we already know how to effectively prevent and cure TB through the half a century of development and progress, there are still more than 1.6 million people who died of TB. In 2014, TB killed 1.5 million people (1.1 million HIV-negative and 0.4 million HIV-positive). The toll comprised 890 000 men, 480 000 women, and 140 000 children. India, Indonesia, and China had the largest number of cases: 23%, 10%, and 10% of the global total, respectively [1]. TB is caused by* Mycobacterium tuberculosis* and spread via air-borne droplets from a cough or sneeze. The majority of infected individuals never develop TB, and only few people would induce active TB.

Bovine tuberculosis (BTB) is zoonotic infectious disease that is by the OIE (Office International des Epizooties) classified as class B animal epidemics. Infected cattle can act as the primary source of infection in other animals and humans. The main route of transmission is the respiratory and digestive tract. Healthy people and animals will be infected by contacting infected animals or drinking their raw milk [2, 3].

BTB is caused by* Mycobacterium bovis* and* Mycobacterium tuberculosis*. BTB is a major infection of work cattle and cows. Most of high-yielding dairy cows and young cattle are infected by BTB [4]. After being infected with TB, cows will decrease milk production and working cattle become emaciated; finally, most infected cattle died of heart failure. BTB not only restricts the development of the livestock industry but also threatens people's health. It has become a worldwide public health problem [5, 6].

BTB has a very long history in Xinjiang, and it has a wide popularity and has a serious impact on the animal husbandry in Xinjiang. According to Xinjiang related archives and records, livestock infection of TB existed in Xinjiang before the founding of China [7]. After the founding of China, Xinjiang quarantined bovine tuberculosis in the early 1950s. Quarantine was via conjunctival sac or use the intradermal allergic reaction method. All positive cows detected in the country documents or policy specific requirements are to be slaughtered. From 1990 to 2007, a total of 1098651 head of cattle were quarantined in Xinjiang; positive rate was 0.88%. But because of the shortage of the subsidy funds, only a part of positive cattle was slaughtered [6].

Mathematical model is the important tool to measure control strategies against various infectious diseases [8]. Mathematical models have played a significant role in understanding the complexity of TB transmission dynamics. The original mathematical models for TB were developed by Blower et al. in 1995 [8]. They established a simple model and a complex model to explain the spread of TB in the population. They demonstrated that it takes one to several hundred years for a TB epidemic to rise, fall, and reach a stable endemic level. Since then, a large number of mathematical models have been created for tuberculosis [9–18]. Blower et al. introduced chemoprophylaxis and treatment in previous models due to drug sensitivity and drug resistance expansion [9]. They concluded that, in order to control TB, treatment failure rates must be lower in developing countries than in developed countries.

Although many studies of dynamical TB models spread between humans have been reported, little work has been performed on such models spread between humans and animals up to now. The purpose of this paper is to propose a TB model between humans and cows to investigate the BTB epidemic situation and analyze the effect of current control strategies in Urumqi. In this paper, based on the reference of the literature, exploring TB transmission mechanism between humans and cows, the dynamic model is established.

The paper is organized as follows. In Section 2, we introduce the data sources. The model establishing and analysis were shown in Section 3, including the calculation of the basic reproductive number and the discussion of positive equilibrium points. The parameter estimation and sensitivity analysis of the model were carried out in Section 4. A discussion is given in Section 5.

## 2. Data Sources

This paper used data from the human and livestock TB epidemiological investigation in Urumqi [19]. The epidemiological investigation was to find the rules and characteristics between human and livestock TB in Urumqi and control the spread of TB better.

### 2.1. Object and Method

#### 2.1.1. Object

We targeted 14 large-scale dairy farms and 8 counties of grazed cows in total of 82271 cows in Urumqi.

#### 2.1.2. Method

(i) Bovine tuberculin intradermal allergy: it is according to *⟪*The Animal Tuberculosis Diagnosis Technology (GB/T 18645-2002)*⟫*.
(ii) The EU intradermal allergic reaction: it is the same as the bovine PPD (purified protein derivative) allergic reaction test. On the other side of the cow neck, injected imported bovine type PPD and avian type PPD at the same time, located between 12 cm and 15 cm doses, were 3000 IU/head and 2500 IU/head. The results of the experiments were measured by the same person before and after injection.
(iii) Interferon-*γ*:
  (1) antigen stimulation: for each cow, 5 mL heparin anticoagulant blood was collected and transported to the laboratory within 30 h at room temperature. Each sample of heparin anticoagulant blood 1.5 mL was taken and injected to three different holes. The 100 gL bovine type PPD, avian type PPD, and negative control phosphate buffer solution (PBS) were taken and joined to heparin anticoagulant blood, respectively, thoroughly mixing after being incorporated into containing 5% CO_2_ incubator in 37°C for 16 h. With Transferpettor absorbing the supernatant, the supernatant was transferred to centrifuge tube (1.5 mL). Namely, it is to stimulate the interferon-*γ* in the supernatant;
  (2) cattle interferon-*γ* enzyme-linked immune sorbent assay (ELISA): For cetuximab coated plate on each hole by adding 50 *µ*L sample dilution liquid, then adding 50 *µ*L measured samples or control, mixing at room temperature 1 h, washing each hole to join 100 *µ*L enzyme labeled antibodies at room temperature for 1 h, and washing after each hole joining 100 *µ*L substrate at room temperature, avoiding light for 30 min after adding stop solution, the OD value of bovine type PPD stimulation supernatants minus OD value of PBS supernatant is more than or equal 0.1, and OD value of bovine type PPD stimulation supernatants minus OD value of avian PPD stimulation supernatants is more than or equal 0.1 is positive, otherwise negative.

### 2.2. Result

#### 2.2.1. The Cow TB Quarantine Results

A total of 82271 cows were quarantined using tuberculin (PPD) intradermal allergic reaction for 8 years in Urumqi of 14 large-scale dairy farms and 8 counties (see Table 1). Results: the result shows that there are 333 positive cows in quarantined cows, so positive rate is 0.40%. For 14 large-scale dairy farms, 35634 cows were quarantined, and the positive rate was 0.51%. Large-scale dairy farm from 2007 to 2014 TB positive rates were 0.37%, 0.64%, 0.55%, 0.52%, 1.55%, 0.09%, 0.15%, and 0.18% (see Table 2). For eight counties in Urumqi, in the cows of scattered households, 46637 cows were quarantined, and the positive rate was 0.32%. The positive rates were 0.06%, 0.63%, 0.17%, 0.32%, 0.60%, 0.23%, 0.14%, and 0% (see Table 3). The positive rate of cows of scattered households was lower than large-scale dairy farm.

#### 2.2.2. Comparison Results of Different BTB Quarantine Method

Use the comparison of the allergy test and *γ*-interferon test of developed countries to test 124 cows of which 199 TB positive samples use the domestic neck allergy quarantine. The results were as follows: domestic PPD intradermal allergy and abroad allergy coincidence rate was 68.59% and interferon-*γ* detection of coincidence rate was 79%. Abroad allergy and interferon-*γ* detection coincidence rate was 86.4%.

#### 2.2.3. Different Methods of TB Quarantine Compared with Pathological Autopsy Results

Domestic pure neck allergy quarantine, abroad allergy test, interferon-*γ* test, and pathologic autopsy results compared with positive coincidence rate are as follows: it was observed that the coincidence rate between the lesion and *γ*-interferon detection was 96.2% and the coincidence rate with the foreign comparative allergy was 92.3%. Test proved that interferon-*γ* compared with abroad allergy was of strong specificity.

#### 2.2.4. Urumqi Cow TB Bacteria Isolation and Identification Results

Harbin Veterinary Research Institute in China makes for the submission of material disease isolated 42 strains of acid-fast bacteria. Among them are already identified 12 strains of* Mycobacterium tuberculosis* complex, including 6 strains of* M. tuberculosis* and* Mycobacterium bovis*.* Mycobacterium tuberculosis* complex separation rate was 3.9%; Spoligotyping and VNTR-MIRU classification method of 12 strains of* Mycobacterium tuberculosis* complex isolates genotyping results showed that 12 strains of isolates of tuberculosis bacterium present eight genotypes, and three of them have unique genotype strains. China Animal Health Center was isolated to 20 strains of bacteria from 26 autopsy positive cows. The classification identification of the bacteria of 20 isolated strains showed that there were three epidemic strains of bovine type accounting for 65%, bovine type BCG accounting for 5%, and other mycobacteria accounting for 30%, respectively.

In 2011, Sanlu milk powder caused damage to a lot of people because of toxic ingredients melamine. Dairy industry had a great adverse impact after this point. As a consequence of the not acquired raw milk, farmers sold and slaughtered a large number of cows so that the large number of cows declined sharply.

Therefore, we can get the point estimate and interval estimation of TB positive cows in Urumqi city in 2007–2014 (see Table 4).

## 3. The Transmission Model

### 3.1. Model Formulation

**Figure 1:**
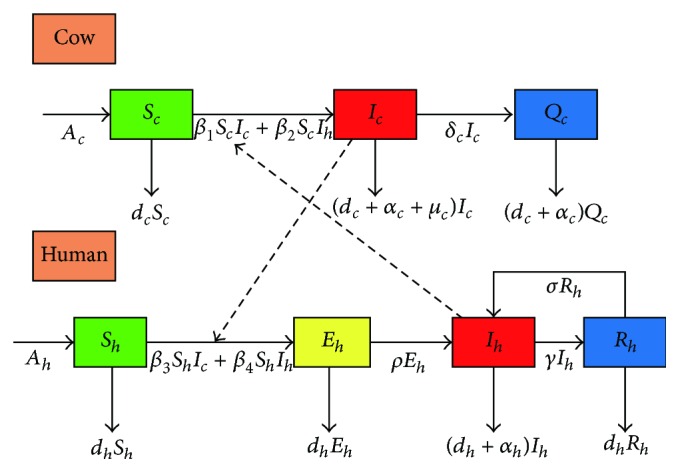
Transmission diagram of TB among humans and cows.

We use a TB model to study the transmission of TB in Urumqi, Xinjiang, China [6, 13, 19]. Model consists of two parts; cow TB model captures the temporal dynamics of three groups: susceptible cows (*S*_*c*_), cows infected with* Mycobacterium tuberculosis* (*I*_*c*_), and cows that are removed after infection with* Mycobacterium tuberculosis* (*Q*_*c*_) (including quarantined and slaughtered cows); human TB model captures the temporal dynamics of four groups: susceptible individuals (*S*_*h*_), latently infected individuals (*E*_*h*_), active infectious TB cases (*I*_*h*_), and recovered (*R*_*h*_). The transmission flow among humans and cows is illustrated in Figure 1.

The model is described by the following system of seven ordinary differential equations:

(1) dScdt=Ac−β1ScIc−β2ScIh−dcSc,dIcdt=β1ScIc+β2ScIh−dc+αc+μcIc−δcIc,dQcdt=δcIc−dc+αcQc,dShdt=Ah−β3ShIc−β4ShIh−dhSh,dEhdt=β3ShIc+β4ShIh−ρEh−dhEh,dIhdt=ρEh−γIh−dh+αhIh+σRh,dRhdt=γIh−σRh−dhRh.

The parameters of the model are explained below: *A*_*c*_ is recruiting of susceptible cows; *d*_*c*_ is natural death rate of cows; *β*_1_ is the rate of cows infected TB via cows; *β*_2_ is the rate of cows infected TB via humans; *α*_*c*_ is mortality rate due to TB of cows; *μ*_*c*_ is the slaughter rate to infected cows; *δ*_*c*_ is the quarantine rate to infected cows; *A*_*h*_ is recruiting of susceptible humans; *d*_*h*_ is the removal rate of livestock workers in dairy farm; *β*_3_ is the rate of humans infected TB via cows; *β*_4_ is the rate of humans infected TB via humans; *ρ* is the progression rate to TB; *α*_*h*_ is mortality rate due to TB of humans; *γ* is the cure rate to TB; *σ* is the rate of relapse to active TB.

### 3.2. Model Analysis

Notice that *Q*_*c*_ is independent of the first six equations, and we start by considering the first six equations:

(2) dScdt=Ac−β1ScIc−β2ScIh−dcSc,dIcdt=β1ScIc+β2ScIh−dc+αc+μcIc−δcIc,dShdt=Ah−β3ShIc−β4ShIh−dhSh,dEhdt=β3ShIc+β4ShIh−ρEh−dhEh,dIhdt=ρEh−γIh−dh+αhIh+σRh,dRhdt=γIh−σRh−dhRh.

Simple algebraic calculation shows that model (2) always has a unique disease-free equilibrium *E*_0_(*A*_*c*_/*d*_*c*_, 0, *A*_*h*_/*d*_*h*_, 0,0, 0). According to the concepts of next generation matrix and reproduction number presented in [24, 25], we define

(3) F=β1ScIc+β2ScIhβ3ShIc+β4ShIh0,V=dc+αc+μcIc+δcIcρEh+dhEhγIh−σRh+dh+αhIh−ρEh.

Noting that the disease-free equilibrium of model (2) is *E*_0_, then

(4) F=β1Sc0β2Scβ3Sh0β4Sh000,V=dc+αc+μc+δc000ρ+dh00−ργ+dh+αh.

Hence, the next generation matrix is

(5) $$\begin{array}{c} FV^{-1}=\left(\begin{array}{ccc} \frac{\beta_{1}A_{c}}{d_{c}\left(d_{c}+\alpha_{c}+\mu_{c}+\delta_{c}\right)} & \frac{\beta_{2}A_{c}\rho }{d_{c}\left(\rho +d_{h}\right)\left(\gamma +d_{h}+\alpha_{h}\right)} & \frac{\beta_{2}A_{c}}{d_{c}\left(\gamma +d_{h}+\alpha_{h}\right)}\\ \frac{\beta_{3}A_{h}}{d_{h}\left(d_{c}+\alpha_{c}+\mu_{c}+\delta_{c}\right)} & \frac{\beta_{4}A_{h}\rho }{d_{h}\left(\rho +d_{h}\right)\left(\gamma +d_{h}+\alpha_{h}\right)} & \frac{\beta_{4}A_{h}}{d_{h}\left(\gamma +d_{h}+\alpha_{h}\right)}\\ 0 & 0 & 0 \end{array}\right). \end{array}$$

The basic reproduction number is given by *ρ*(*FV* ^−1^) and

(6) R0=−a+a2−4b2,a=−β1Acdcdc+αc+μc+δc−β4Ahρdhρ+dhγ+dh+αh,b=β1Acdcdc+αc+μc+δcβ4Ahρdhρ+dhγ+dh+αh−β2Acρdcρ+dhγ+dh+αhβ3Ahdhdc+αc+μc+δc.

According to the conclusions of the literature [24, 25], the following results are obtained.

**Theorem 1 .**

When *R*_0_ < 1, *E*_0_ is local stable; when *R*_0_ > 1, *E*_0_ is unstable.

Using a similar argument as in the proof of proposition  3.3 in [26], we can show that when *R*_0_ > 1, model (2) has at least one endemic equilibrium *E* ^*∗*^. On the stability of the endemic equilibrium, one has the following theorem.

**Theorem 2 .**

Assume that *R*_0_ > 1; the endemic equilibrium *E* ^*∗*^ is globally asymptotically stable.

**Proof**

Let

(7) V1=Sc−Sc∗−Sc∗ln⁡ScSc∗+Ic−Ic∗−Ic∗ln⁡IcIc∗,V2=Sh−Sh∗−Sh∗ln⁡ShSh∗+Eh−Eh∗−Eh∗ln⁡EhEh∗,V3=Ih−Ih∗−Ih∗ln⁡IhIh∗,V4=Rh−Rh∗−Rh∗ln⁡RhRh∗.

Differentiating *V*_*i*_  (*i* = 1,2, 3,4) along the solutions of model (2), then

(8) V1′1−Sc∗ScAc−β1ScIc−β2ScIh−dcSc+1−Ic∗Ic·β1ScIc+β2ScIh−dc+αc+μc+δcIc.

Further, using the equilibrium satisfying equations, we have

(9) V1′−dcSc−Sc∗2Sc+β1Sc∗Ic∗1−Sc∗Sc1−ScIcSc∗Ic∗+β2Sc∗Ih∗1−Sc∗Sc1−ScIhSc∗Ih∗+β1Sc∗Ic∗1−Ic∗IcScIcSc∗Ic∗−IcIc∗+β2Sc∗Ih∗1−Ic∗IcScIhSc∗Ih∗−IcIc∗=−dcSc−Sc∗2Sc+β1Sc∗Ic∗1−ScIcSc∗Ic∗−Sc∗Sc+IcIc∗+β2Sc∗Ih∗1−ScIhSc∗Ih∗−Sc∗Sc+IhIh∗+β1Sc∗Ic∗ScIcSc∗Ic∗−IcIc∗−ScSc∗+1+β2Sc∗Ih∗ScIhSc∗Ih∗−IcIc∗−IhScIc∗Ih∗Sc∗Ic+1=−dcSc−Sc∗2Sc+β1Sc∗Ic∗2−Sc∗Sc−ScSc∗+β2Sc∗Ih∗2−Sc∗Sc+IhIh∗−IcIc∗−IhScIc∗Ih∗Sc∗Ic≤β2Sc∗Ih∗IhIh∗−IcIc∗−ln⁡IhIh∗+ln⁡IcIc∗.

Through the same calculation, we obtain

(10) V2′1−Sh∗ShAh−β3ShIc−β4ShIh−dhSh+1−Eh∗Ehβ3ShIc+β4ShIh−ρ+dhEh=−dhSh−Sh∗2Sh+β3Sh∗Ic∗1−Sh∗Sh1−ShIcSh∗Ic∗+β4Sh∗Ih∗1−Sh∗Sh1−ShIhSh∗Ih∗+β3Sh∗Ic∗1−Eh∗EhShIcSh∗Ic∗−EhEh∗+β4Sh∗Ih∗1−Eh∗EhShIhSh∗Ih∗−EhEh∗≤β3Sh∗Ic∗1−ShIcSh∗Ic∗−Sh∗Sh+IcIc∗+β4Sh∗Ih∗1−ShIhSh∗Ih∗−Sh∗Sh+IhIh∗+β3Sh∗Ic∗ShIcSh∗Ic∗−EhEh∗−IcShEh∗Ic∗Sh∗Eh+1+β4Sh∗Ih∗ShIhSh∗Ih∗−EhEh∗−IhShEh∗Ih∗Sh∗Eh+1=β3Sh∗Ic∗2−Sh∗Sh+IcIc∗−EhEh∗−IcShEh∗Ic∗Sh∗Eh+β4Sh∗Ih∗2−Sh∗Sh+IhIh∗−EhEh∗−IhShEh∗Ih∗Sh∗Eh≤β3Sh∗Ic∗IcIc∗−EhEh∗−ln⁡IcIc∗+ln⁡EhEh∗+β4Sh∗Ih∗IhIh∗−EhEh∗−ln⁡IhIh∗+ln⁡EhEh∗,V3′1−Ih∗IhρEh+σRh−dh+αh+γIh=ρEh∗1−Ih∗IhEhEh∗−IhIh∗+σRh∗1−Ih∗IhRhRh∗−IhIh∗≤ρEh∗EhEh∗−IhIh∗−ln⁡EhEh∗+ln⁡IhIh∗+σRh∗RhRh∗−IhIh∗−ln⁡RhRh∗+ln⁡IhIh∗.

Similarly, it is easy that

(11) V4′1−Rh∗RhγIh−dhRh=γIh∗1−Rh∗RhIhIh∗−RhRh∗≤γIh∗IhIh∗−RhRh∗−ln⁡IhIh∗+ln⁡RhRh∗.

Now, construct the following Lyapunov function:

(12) L=ργβ3Sh∗Eh∗Ic∗V1+ργβ2Eh∗Ih∗Sc∗V2+γβ2β4Sh∗Ih∗2Sc∗+γβ2β3Sh∗Ih∗Sc∗Ic∗V3+σβ2β4Sh∗Ih∗Rh∗Sc∗+σβ2β3Sh∗Rh∗Sc∗Ic∗V4.

Then,

(13) L˙≤ργβ3Sh∗Eh∗Ic∗β2Sc∗Ih∗IhIh∗−IcIc∗−ln⁡IhIh∗+ln⁡IcIc∗+ργβ2Eh∗Ih∗Sc∗β3Sh∗Ic∗IcIc∗−EhEh∗−ln⁡IcIc∗+ln⁡EhEh∗+ργβ2Eh∗Ih∗Sc∗β4Sh∗Ih∗IhIh∗−EhEh∗−ln⁡IhIh∗+ln⁡EhEh∗+γβ2β4Sh∗Ih∗2Sc∗ρEh∗EhEh∗−IhIh∗−ln⁡EhEh∗+ln⁡IhIh∗+γβ2β4Sh∗Ih∗2Sc∗σRh∗RhRh∗−IhIh∗−ln⁡RhRh∗+ln⁡IhIh∗+γβ2β3Sh∗Ih∗Sc∗Ic∗ρEh∗EhEh∗−IhIh∗−ln⁡EhEh∗+ln⁡IhIh∗+γβ2β3Sh∗Ih∗Sc∗Ic∗σRh∗RhRh∗−IhIh∗−ln⁡RhRh∗+ln⁡IhIh∗+σβ2β4Sh∗Ih∗Rh∗Sc∗γIh∗IhIh∗−RhRh∗−ln⁡IhIh∗+ln⁡RhRh∗+σβ2β3Sh∗Rh∗Sc∗Ic∗γIh∗IhIh∗−RhRh∗−ln⁡IhIh∗+ln⁡RhRh∗≤0.

It can be verified that the largest invariant set where *L*′ = 0 is singleton *E* ^*∗*^. Therefore, by LaSalle's invariance principle, *E* ^*∗*^ is globally asymptotically stable.

## 4. Model Application

### 4.1. Parameter Estimation

The values of parameters for model (1) are listed in Table 5. According to the national policy, the positive livestock infected TB should be slaughtered, however, due to the lack of funds and the nontimely payment of the slaughter of livestock, resulting in the fact that TB positive livestock are not completely slaughtered. So we choose *μ*_*c*_ = 0.85 and *δ*_*c*_ = 0.12.

We use 2007–2014 in Urumqi dairy herds number and positive rate data to estimate the parameters of the model; we estimate that the initial condition of infected cows is *I*_*c*_(0) = 59. The other initial conditions are assumed to be *S*_*c*_(0) = 21000, *Q*_*c*_(0) = 70, *S*_*h*_(0) = 800, *E*_*h*_(0) = 100, *I*_*h*_(0) = 30, and *R*_*h*_(0) = 20, respectively.

The parameters *β*_1_, *β*_2_, and *β*_3_ are obtained by fitting the model to data. We ignored humans infected TB via humans; hence, we make *β*_4_ = 0. By least-square fitting and Bootstrap method, we can obtain the point estimation and confidence interval for transmission coefficient which are listed in Table 6, respectively.

**Figure 2:**
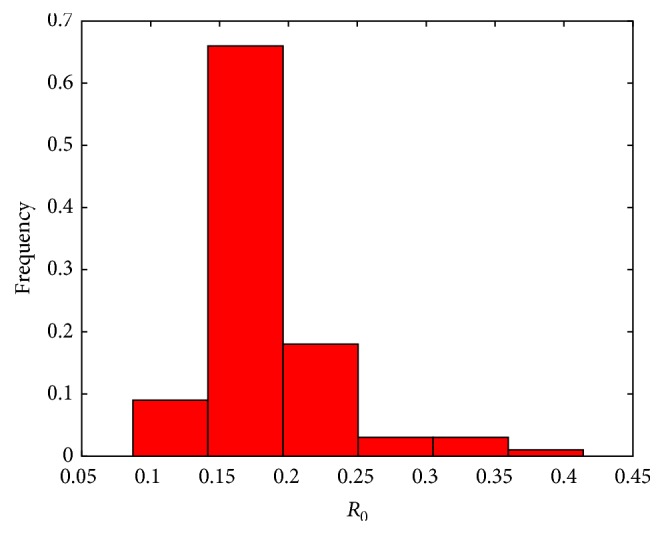
The frequency histogram for *R*_0_.

**Figure 3:**
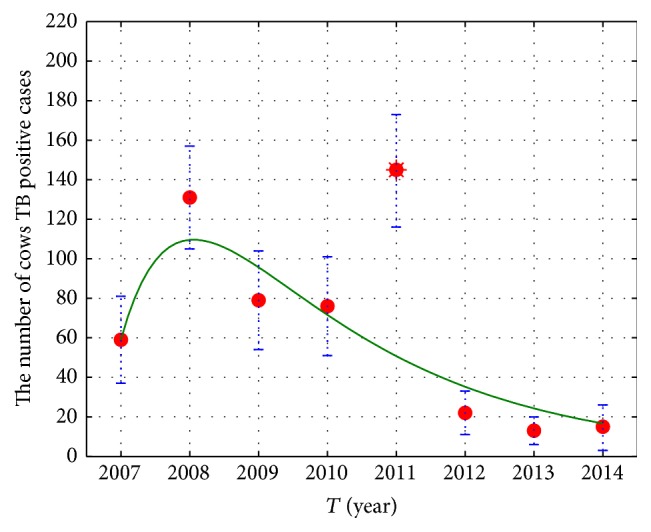
The cow TB positive fitting model in 2007–2014.

**Figure 4:**
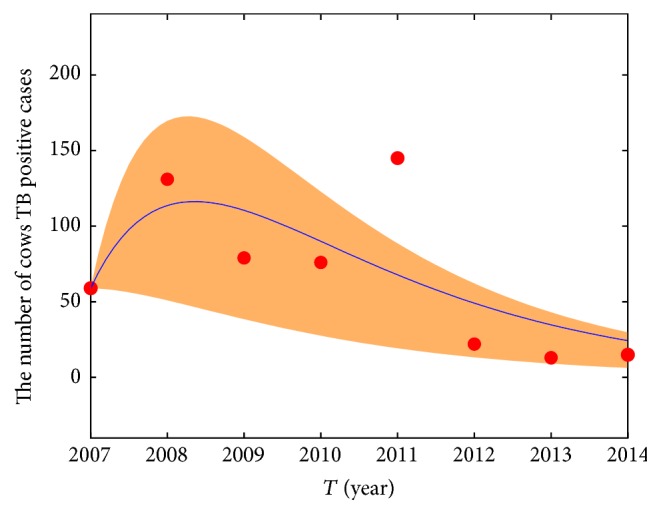
The cow TB positive fitting model for Bootstrap in 2007–2014.

**Figure 5:**
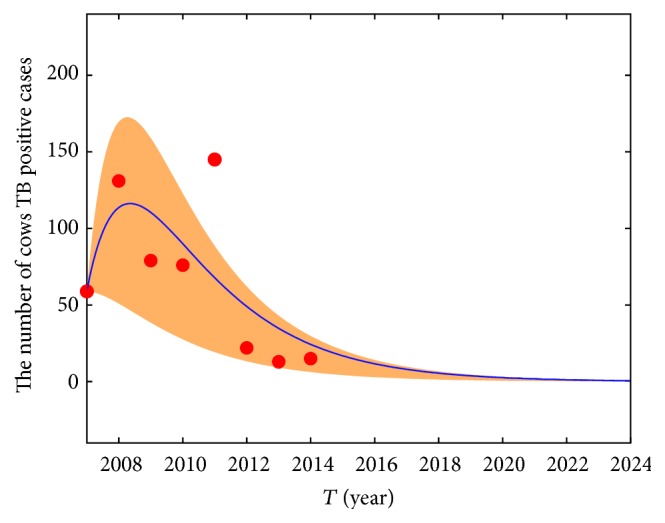
The tendency of the number of cow positive TB cases.

Based on Table 6, we obtained the basic reproduction number *R*_0_ ≈ 0.1811. The result shows that disease will not break out under current situation by Theorem 1. We give a histogram of *R*_0_ obtained by using the Bootstrap method (see Figure 2). In 2011, some of the large-scale dairy farm owners changed and the new buy cows from other places, so the rate of TB positive cows is very high. We regard this point as outlier. We discard this point, estimate the number of TB positive cows in 2007–2014, and draw the 95% confidence interval (see Figure 3). The result shows that the fitting effect is good; in fact, we estimate the number of TB positive cows in 2007–2014 and provide the confidence belt by all of the data (see Figure 4). We can predict the general tendency of the epidemic according to the current situation, which is presented in Figure 5. The prediction shows that disease will vanish around 2020 (see Figure 5).

### 4.2. Sensitivity Analysis

For the sensitivity analysis, Latin hypercube sampling was used to sample parameters that appear in the derived expression for basic reproduction number *R*_0_. Uncertainty and sensitivity analysis based on Latin hypercube sampling has been previously applied to disease transmission models. Thus, in order to examine the sensitivity of our results to parameter variations, we use Latin hypercube sampling to examine the dependence of basic reproduction number *R*_0_.

**Figure 6:**
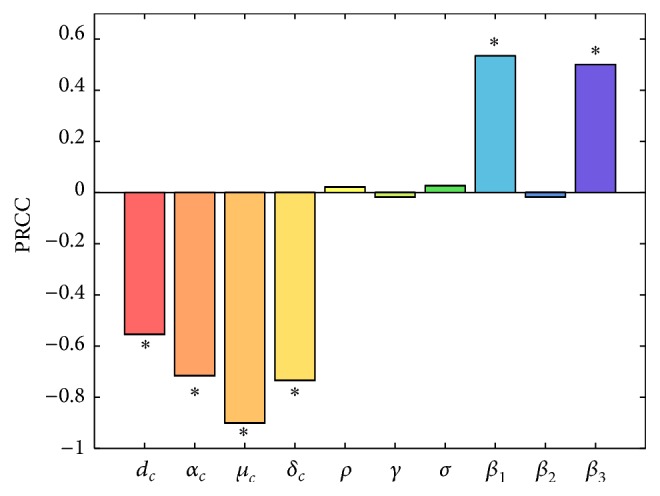
Partial rank correlation coefficients (PRCCs) result for the dependence of *R*_0_ on each parameter.

We choose sample size *n* = 2000, parameters of interest as the input variables, and the value of *R*_0_ as the output variable. The PRCC values of ten parameters are listed in Table 7 and shown in Figure 6. The ordering of these PRCCs corresponds to the level of statistical influence the parameter has on the variability for the basic reproduction number *R*_0_. The larger the PRCCs in absolute value, the more important the parameter in responding to the change in *R*_0_. Plus sign or minus sign means the influence is positive or negative, respectively. Figure 6 shows that *β*_1_ and *β*_3_ have positive impact upon *R*_0_, whilst *d*_*c*_, *α*_*c*_, *μ*_*c*_, and *δ*_*c*_ have negative impact. We also know that *R*_0_ is not sensitive to parameters *ρ*, *γ*, *σ*, and *β*_2_.

Table 7 shows that the slaughter rate to infected cows *μ*_*c*_(|PRCC| = 0.9008) has the greatest impact on *R*_0_. Then, the quarantine rate *δ*_*c*_(|PRCC| = 0.7349) to infected cows has the greater impact on *R*_0_. Hence, from sensitivity and mathematical analysis, we conclude that the most effective approach to reduce the TB infection is to increase parameters *μ*_*c*_ and *δ*_*c*_.

**Figure 7:**
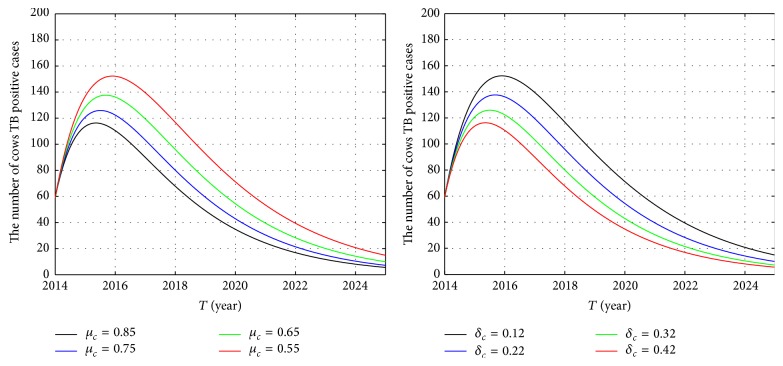
The influence of parameters *μ*_*c*_ and *δ*_*c*_ on the number of cow positive TB cases.

In the following, we focus on parameters *μ*_*c*_ and *δ*_*c*_. The influence of parameters *μ*_*c*_ and *δ*_*c*_ on the number of cows TB positive cases is shown in Figure 7. We can see from Figure 7 that, with the increase in slaughter rate, the positive rate of TB in dairy cows will be greatly reduced. Similarly, this phenomenon is also reflected in the effect of quarantine rate on the number of TB positive cows; appropriate increase of the quarantine rate of TB positive cows can also be a good control of the spread of TB.

It is very significant to investigate the effect of slaughter rate and quarantine rate on basic reproduction number *R*_0_. Due to the lack of funds and the nontimely payment of the slaughter of cattle, resulting in the fact that TB positive cattle are not completely slaughtered, when the slaughter rate can not reach a high proportion of cases, appropriate improvement to the quarantine of sick cattle can also control the epidemic of BTB.

## 5. Discussion

TB infection exists widely in the world. In Xinjiang, TB is one of the major infectious diseases that seriously endanger the health of people. Xinjiang is one of the large pastoral areas in China. The prevalence of BTB not only restricts the development of the livestock industry in Xinjiang but also threatens people's health. To investigate the prevalence of BTB in Urumqi, a total of 82271 cows in Urumqi areas from 14 large-scale dairy farms and 8 counties of grazed cows were quarantined [19]. We establish a dynamical model for TB of humans and cows. We get the disease-free equilibrium point, discuss the positive equilibrium point, estimate the parameters, and conduct the sensitivity analysis. The sensitivity coefficients (PRCCs) of the parameters with respect to the basic reproduction number are shown in Figure 6. The results indicate that the slaughter rate and quarantine rate are the main factors affecting the spread of BTB, so the standard slaughter and quarantine management of the TB positive cows will inhibit the spread of BTB effectively. The simulation results reveal the main trend of BTB epidemic in Urumqi and also a prediction for the trend of the BTB infection. In 2011, some of the large-scale dairy farm owners changed and the new brought cows from other places, so the rate of TB positive cows was very high. This point has a little impact on our fitting effect. Finally, we predict the number of TB positive cows in Urumqi from 2014 to 2024. Figure 5 shows that the number of TB positive cows will be close to zero in our model. According to recent epidemiological investigation, BTB effective control had been obtained in Urumqi. The result shows that the current control measures are effective.

## Figures and Tables

**Table 1:** The cow TB quarantine statistics in 2007–2014.

Time	Cow herds (head)	TB quarantine (head)	Positive (head)	Positive rate (%)
2007	21232	10084	28	0.28%
2008	20789	15359	97	0.63%
2009	24527	12099	39	0.32%
2010	20789	9543	35	0.37%
2011	15066	10304	99	0.96%
2012	13211	9071	15	0.17%
2013	9429	9830	14	0.14%
2014	14638	5981	6	0.10%

Total	139681	82271	333	0.40%

**Table 2:** The cow TB quarantine statistics of scale cow field in 2007–2014.

Time	TB quarantine (head)	Positive (head)	Positive rate (%)
2007	6955	26	0.37%
2008	6732	43	0.64%
2009	4865	27	0.55%
2010	2155	11	0.52%
2011	3926	61	1.55%
2012	4263	4	0.09%
2013	3418	5	0.15%
2014	3360	6	0.18%

Total	35634	183	0.51%

**Table 3:** The cow TB quarantine statistics of grazed cows in 2007–2014.

Time	TB quarantine (head)	Positive (head)	Positive rate (%)
2007	3129	2	0.06%
2008	8627	54	0.63%
2009	7234	12	0.17%
2010	7428	24	0.32%
2011	6378	38	0.60%
2012	4808	11	0.23%
2013	6412	9	0.14%
2014	2621	0	0%

Total	46637	150	0.32%

**Table 4:** The point estimate and interval estimation of TB positive cows in Urumqi city in 2007–2014.

Time	Cow herds (head)	Positive rate (%)	Point estimate	95% CI
2007	21232	0.28%	59	[37,81]
2008	20789	0.63%	131	[105,157]
2009	24527	0.32%	79	[54,104]
2010	20789	0.37%	76	[51,101]
2011	15066	0.96%	145	[116,173]
2012	13211	0.17%	22	[11,33]
2013	9429	0.14%	13	[6,20]
2014	14638	0.10%	15	[3,26]

**Table 5:** Descriptions and values of parameters in model.

Parameter	Value	Interpretation	Source
*A*_*c*_	3853.8	Recruiting of susceptible cows	[19]
*d*_*c*_	1/5	Natural death rate of cows	Estimation
β_1_	1.0995 × 10^−5^	The rate of cows infected TB via cows	Fitting
β_2_	5.7803 × 10^−5^	The rate of cows infected TB via humans	Fitting
α_*c*_	0	Mortality rate due to TB of cows	Estimation
μ_*c*_	0.85	The slaughter rate to infected cows	Estimation
δ_*c*_	0.12	The isolation rate to infected cows	Estimation
*A*_*h*_	36	Recruiting of susceptible humans	[20–23]
*d*_*h*_	0.04	The removal rate of livestock worker in dairy farm	[20–23]
β_3_	1.6252 × 10^−5^	The rate of humans infected TB via cows	Fitting
β_4_	0	The rate of humans infected TB via humans	Estimation
ρ	1/3	Progression rate to TB	[8]
α_*h*_	0.139	Mortality rate due to TB of humans	[8]
γ	0.058	Cure rate to TB	[8]
σ	0.01	Rate of relapse to active TB	[8]

**Table 6:** The point estimation and 95% Bootstrap confidence interval for the parameters and *R*_0_.

Parameter	Point estimate	95% Bootstrap CI
β_1_	1.0995 × 10^−5^	[7.49 × 10^−6^, 1.71 × 10^−5^]
β_2_	5.7803 × 10^−5^	[2.72 × 10^−5^, 9.53 × 10^−5^]
β_3_	1.6252 × 10^−5^	[1.76 × 10^−19^, 4.45 × 10^−18^]
*R*_0_	0.1811	[0.123,0.281]

**Table 7:** Partial rank correlation coefficients (PRCCs) for aggregate *R*_0_ and each input parameter.

Input parameter	PRCC	*p* value
*d*_*c*_	−0.5549	0
α_*c*_	−0.7175	0
μ_*c*_	−0.9008	0
δ_*c*_	−0.7349	0
ρ	0.0223	0.3210
γ	−0.0187	0.4046
σ	0.0270	0.2293
β_1_	0.5341	0
β_2_	−0.0177	0.4292
β_3_	0.5001	0
